# Tracking pathogen-related markers with eDNA in natural areas: how environmental factors shape surveillance strategies

**DOI:** 10.1186/s13567-026-01746-6

**Published:** 2026-04-28

**Authors:** Alberto Perelló, Camilla Smoglica, Carlos González-Crespo, Marta Pérez-Sancho, David González-Barrio, Carmen Herranz, Alejandro Dashti, Sergio Sánchez, David Carmena, Amir Reza Varzandi, Ezio Ferroglio, Lucas Domínguez, Beatriz Martínez-López, Christian Gortázar

**Affiliations:** 1 SaBio, Instituto de Investigación en Recursos Cinegéticos (IREC) CSIC-UCLM-JCCM, 13071 Ciudad Real, Spain; 2 Department of Veterinary Medicine, University of Teramo, 64100 Teramo, Italy; 3 Center for Animal Disease Modeling and Surveillance, School of Veterinary Medicine, University of California-Davis, Davis, CA USA; 4 VISAVET Health Surveillance Center, Complutense University of Madrid, 28040 Madrid, Spain; 5 Department of Animal Health, Faculty of Veterinary, Complutense University of Madrid, Madrid, Spain; 6 Parasitology Reference and Research Laboratory, Spanish National Centre for Microbiology, Madrid, Spain; 7 Centre for Biomedical Research Network in Infectious Diseases (CIBERINFEC), Health Institute Carlos III, 28029 Madrid, Spain; 8 Department of Veterinary Sciences, School of Agriculture and Veterinary Medicine, University of Turin, Turin, Piedmont Italy

**Keywords:** Environmental DNA, zoonosis, disease surveillance, non-invasive, integrated wildlife monitoring, episystem, natural areas

## Abstract

The wildlife-livestock-environment interface is a complex system with implications for biodiversity and diseases. Environmental nucleic acid detection (ENAD) is a non-invasive method for monitoring DNA/RNA from microorganisms, meiofauna, and hosts. However, how environmental variables influence ENAD in heterogeneous geographic contexts remains poorly explored. In this study, 18 sites were evaluated in Iberian Peninsula, collecting 10 surface sponge samples per site and a total of 146 environmental fecal samples to investigate how environmental, mammal community and wildlife health variables may influence ENAD, using different pathogen-related markers as the study model. Differences in ENAD were assessed among sponge sampling methods and between sponge and fecal samples. The results show that environmental characteristics influence ENAD, at least for pathogen-related markers and at larger geographic scales. Greater pathogen marker diversity and richness was observed at higher latitudes. Most markers found in feces were also detectable in surface sponges. Combining different sponge sampling methodologies provided the best overall coverage of detectable environmental DNA. A predictive map linking ENAD in sponges to environmental factors was developed.

**Supplementary Information:**

The online version contains supplementary material available at 10.1186/s13567-026-01746-6.

## Introduction

The wildlife-livestock-environment interface is a complex system with profound implications for biodiversity conservation and microorganism circulation [1, 2]. Biodiversity and disease dynamics are influenced by multiple factors, including pathogen specificity, transmission routes, and habitat characteristics [3]. Current literature presents two key hypotheses regarding the interaction between biodiversity and pathogens: the amplification effect and the dilution effect, both of which vary based on the pathogens involved and the geographic regions [4, 5]. In this context, it is crucial to explore the roles that wildlife and livestock play in pathogen maintenance within the environment, based on the concept known as episystem. This approach integrates the interactions between pathogens, hosts, and environmental factors across specific spatial and temporal scales [6]. The development and validation of non-invasive monitoring methodologies to assess biodiversity and pathogen dynamics on the wildlife-livestock-environment interface could be a crucial tool for the implementation of integrated surveillance programs.

In this framework, environmental nucleic acid detection (ENAD) emerges as a powerful tool to monitor a broad range of organisms, from microbes (bacteria, viruses, protists) to macroorganisms [7]. To date, ENAD has primarily been applied in aquatic environments, using PCR, qPCR, or metabarcoding, particularly focused on pathogen monitoring, including those introduced or spread by invasive species [7]. Its potential has been recognized by the World Organization for Animal Health (WOAH), which includes ENAD in its guidelines for detecting *Gyrodactylus salaris* in salmonids [8]. Recent ENAD surveys have yielded highly promising results for monitoring terrestrial mammals and their associated pathogens [9]. For example, ENAD methodologies have been applied to detect African swine fever virus (ASFV) from turbid water and soil samples, as well as ASFV in pig breeding facilities or *Mycobacterium tuberculosis* complex (MTC) in cattle farms and European bison populations using dry sponges pre-hydrated with a specific surfactant applied to the animals' skin and contaminated surfaces [10–13]. These sponges have also recently been used to detect and monitor pathogen markers related to *M. avium* subp. *paratuberculosis*, *Coxiella burnetii*, *Brucella* spp., *Salmonella enterica* and *Escherichia coli* on surfaces at risk points on farms, such as waterers and feeders, and on animals [14, 15]. In addition, ENAD has also been applied to substrates such as equine enrichment toys [16], honey [17], invertebrates [18–20], as well as fecal samples from amphibians, reptiles, and livestock [14–21]. Similarly, ENAD from non-invasive sampling of wildlife feces is recognized as an effective tool for infectious disease surveillance in natural areas providing valuable data for assessing disease circulation, zoonotic risks, and ecosystem health without the need for direct animal handling [22]. Indeed, ENAD offers advantages in terms of sampling efficiency, reduced processing time, and lower costs—particularly in challenging contexts involving elusive or low-density animal populations [7, 23]. When combined with spatial and epidemiological data, ENAD can also offer a comprehensive understanding of pathogen dynamics across a geographic area [7]. For instance, a pilot study on non-invasive monitoring in outdoor farming systems integrated vertebrate richness data and ENAD-derived health markers, revealing a negative correlation between pathogen marker richness and farm vertebrate richness [14]. Similarly, a recent study carried out in Poland demonstrated the application of ENAD for detecting MTC in European bison populations within regions with historical records of tuberculosis [11]. These studies have highlighted the effectiveness of ENAD approaches—based on fecal and sponge sampling integrated with ecological data—in detecting pathogens in both livestock and wild animal populations, whether in outdoor farming environments or natural ecosystems [11, 14]. However, these investigations have been generally limited to single, environmentally homogeneous areas. Consequently, the extent to which environmental variables influence ENAD performance remains poorly explored, particularly in heterogeneous geographic contexts. Exploring this variability is essential to validate ENAD as a robust surveillance tool adaptable to different geographic and ecological conditions.

In this regard, the present study aims to evaluate the relationship between ENAD, mammal community characteristics, and environmental factors across 18 sites on the Iberian Peninsula, using the detection of pathogen-related environmental DNA (eDNA) markers as a proof of concept. Indeed, eDNA from sponges and fecal samples were analyzed for pathogen-specific molecular markers, providing insight into eDNA detection and ENAD influencing factors across heterogeneous geographic areas.

## Materials and methods

### Study area

This work has been developed in 18 pilot monitoring sites throughout the Iberian Peninsula, 15 Spanish and 3 Portuguese, which participated in a pilot network for integrated wildlife monitoring (Wildlife Health Surveillance Plan -WHSP- of Spain) [24, 25]. The sites were selected to ensure they were representative of the five bioregions: Atlantic Spain (*n* = 2); Cereal plains (*n* = 4); Continental Mediterranean ecosystems (*n* = 7); Inland mountains (*n* = 1); and Southern and eastern coast (*n* = 4); recognized in the WHSP of Spain and Portugal [26, 27].

The geographic location of the study sites are the following: (1) Cantabrian Mountains; (2) Llanada alavesa valley; (3) Northern coast; (4) Pre-Pyrenees; (5) Trás-os-montes region; (6) Trás-os-montes region; (7) Catalonia coast (8) Iberian System; (9) Central System; (10) San Pedro Mountains; (11) Toledo Mountains; (12) Guadiana Valley; (13) Campo de Montiel region; (14) Sierra Morena Mountains; (15) Cordillera Bética Mountains; (16) Baixo Alentejo region; (17) North Seville Mountains; (18) Guadalquivir Valley.

### Surface sponges eDNA sampling

Two sponges from surfaces were taken in the 50 m^2^ in front of each of 5 randomly selected camera traps (CTs) of each of the 18 CT grids (180 sponges in total) deployed for wildlife species monitoring purposes (Wildlife Health Surveillance Plan -WHSP- of Spain). In front of each CT, one sponge sampled objects such as stones and tree trunks (O-sponge) and a second sponge sampled the surface of bare soil including feces or rootings if present (S-sponge). A total of ten dry sponges (3M^™^ Dry-Sponge; 3M-España, Madrid, Spain), pre-hydrated with 15 mL of an isotonic surfactant nucleic acid-preserving liquid, were taken per study site [13, 14].

All sampling points within a study site were collected on the same day between 5:30 AM and 10:30 AM during April–June 2023. Sampling was conducted systematically from south to north to ensure consistent conditions across all locations.

### Fecal eDNA sampling

Fecal material was collected along a 200-m transect around each camera trap, resulting in a total transect length of 1 km per study site. Fecal material found was identified as belonging to Eurasian wild boar (*Sus scrofa*) or wild ruminant species (mostly red deer, *Cervus elaphus*, and roe deer, *Capreolus capreolus*). If fresh samples were found, 5–10 g portions collected into sterile bags and preserved refrigerated until reaching the laboratory, where samples were stored frozen at −20 ºC until DNA extraction.

### Nucleic acid extraction

The extraction and purification of environmental DNA (eDNA) from surface sponge samples was performed using the QIAamp Fast DNA Stool Mini Kit (Qiagen Hilden; Germany), starting from the pellet obtained after centrifuging 900 μL of the sample for 3 min at 13 000 rpm [14]. Genomic DNA from fecal samples was isolated from approximately 200 mg of each fecal sample using the same Qiagen kit, in accordance with the manufacturer’s instructions, except for the step where samples were mixed with the InhibitEX buffer, where the incubation time was changed to 10 min at 95 °C. Extracted and purified DNA samples were eluted in 200 μL of PCR-grade water and stored at 4 °C until further molecular analysis.

All sponge and fecal samples were tested for PCR inhibition by including internal control in each PCR reaction, discarding four of 180 sponge samples (2.22%; all soil samples), and seven of 146 fecal samples (4.79%; all of them ruminant samples).

### Molecular detection

The pathogen-realted markers analyzed using PCR techniques (Table 1) include bacteria: *E. coli* (*uidA*, *stx1*, *stx2* and *eae*), MTC (IS*6110* and *mpb*70), *Salmonella* spp. (*invA*), *C. burnetii* (IS*1111*), *Brucella* spp. (IS*711*), *M. avium* subp. *paratuberculosis* (IS*900*); and parasites: *Balantioides coli, Blastocystis sp.*, *Cryptosporidium* spp, *Encephalitozoon* spp, *Enterocytozoon bieneusi, Giardia duodenalis*, and *Toxoplasma gondii*.

**Table 1:** **Pathogen-related molecular targets analysed in this study**

Pathogen	Targeted gene	PCR technique	References
Bacteria			
*E. coli*	*uidA*	Real-time PCR	[28, 29]
Shiga toxin-producing *E. coli*	*stx1, stx2* and *eae*	Real-time PCR	Foodproof STEC Screening Lyokit, Biotecon diagnosis GmbH, Postdam, Germany
*M. tuberculosis* complex	IS*6110* and *mpb*70	Real-time PCR	[30, 31]
*Salmonella* spp.	*invA*	Real-time PCR	[32]
*C. burnetii*	IS*1111*	Real-time PCR	Sponges [33] and feces [34]
*Brucella* spp.	IS*711*	Real-time PCR	[35]
*M. avium* subp. *paratuberculosis*	IS*900*	Real-time PCR	[36]
Parasites			
*B. coli*	ITS1–5.8 s-rRNA–ITS2 region and the *ssu*-rRNA gene	Direct PCR	[37]
*Blastocystis* sp*.*	*ssu* rRNA gene	Direct PCR	[38]
*Cryptosporidium* spp.^a^	*ssu* rRNA gene	Nested PCR	[39]
*E. bieneusi*^b^	*ssu* rRNA gene	RT-PCR	[40, 41]
*Encephalitozoon* spp.^c^	*ssu* rRNA gene	RT-PCR	[40, 42]
*G. duodenalis*^d^	*ssu* rRNA gene	Real-time PCR (qPCR)	[43]
*T. gondii*	200- to 300-fold repetitive 529 bp DNA fragment in the parasite genome	Direct PCR	[44, 45]

^a^Specific subtyping tools targeting the partial 60-kDa glycoprotein gene (gp60) were used in those samples that tested positive for *Cryptosporidium* spp. by *ssu*-PCR for *C. canis* [46].

^b^Samples identified as *E. bieneusi* positive were genotyped using a nested PCR protocol to amplify a fragment of the internal transcribed spacer (ITS) region as well as portions of the flanking large and small subunits of the ribosomal RNA (*ssu* rRNA) gene, as previously described [47].

^c^Samples identified as *Encephalitozoon* spp. positive were genotyped using a nested PCR protocol to amplify the ITS marker as previously described by Katzwinkel-Wladarsch et al. [48], allowing, in turn, the identification of species.

^d^*Giardia*-positive isolates that yielded cycle threshold (CT) values ≤ 34 in RT-PCR were subsequently reassessed by a nested PCR to amplify a fragment of the *ssu* rRNA gene [49, 50] to determine the molecular diversity of the parasite at the assemblage level. Samples that tested positive by *ssu*-PCR were re-amplified at the genes codifying the glutamate dehydrogenase (*gdh*), β-giardin (*bg*) and triose phosphate isomerase (*tpi*) proteins to determine the molecular diversity of the parasite at the sub-assemblage level. A semi-nested PCR was used to amplify a fragment of the *gdh* gene [51] and nested PCRs were used to amplify fragments of the *bg* and *tpi* genes, respectively [52–54].

More details on PCR protocols are specified in Additional file 8 (supplementary methods section).

### Environmental factors influencing pathogen-related marker diversity detected on surfaces in natural areas

#### Sponge molecular markers diversity index

A Shannon diversity index (H’) was calculated to quantify and standardize the diversity of molecular markers detected in sponge samples across study sites. This analysis was performed using the *dplyr* (v1.1.4) and *vegan* (v2.7-1) R packages [55, 56]. For each study area, five sampling points were established, and the abundance value for each molecular marker was determined by the number of positive sampling points detected within each study area (range: 0–5).

#### Study area classification

To investigate the influence of environmental factors on pathogen-related marker diversity detected through ENAD on surfaces in natural areas, study areas were classified using a hierarchical clustering on principal components (HCPC; see Additional file 3). This analysis was implemented using the *FactoMineR* (v2.11) and *factoextra* (v1.0.7) R packages [57, 58].

The optimal number of clusters was determined using a combination of environmental variables (land cover and climatic parameters; see Additional file 4), mammal community characteristics (see Additional file 8—supplementary methods section—for more information on the parameters used), and wildlife health parameters (described in Perelló et al. -manuscript under review-; see Additional file 4) [59]. This classification allowed the comparison of pathogen-related marker diversity (H’) detected in sponge samples and the assessment of factors potentially driving molecular detection in natural areas.

#### Spatial distribution modeling of clusters in the Iberian Peninsula

To predict the spatial distribution of identified clusters across the Iberian Peninsula, a random forest (RF) classification model was performed using the *caret* (v7.0-1) and *randomForest* (v4.7-1.2) packages in R [60, 61]. The model was based on environmental factors (land cover and climatic variables) as predictors and the HCPC-defined clusters as response categories. All analyses were conducted with a fixed random seed to ensure reproducibility.

Model performance was evaluated using a leave-one-out cross-validation (LOOCV), wherein each observation was sequentially used as validation data while the remaining observations served as training data. The cross-validation parameters were established using the *trainControl* function with LOOCV specified as the resampling method. Class probabilities were calculated for each iteration, and predictions were systematically stored for subsequent analysis.

The RF model was trained using the *train* function, with cluster designation as the response variable. Predictor variables incorporated multiple land cover classifications (according to the classifications made by Barroso et al. [24] from Corine LandCover, only shrubland, forest coverage, bare land, grassland and urban land uses were taken into account) [24, 62] and selected bioclimatic variables established by the United States Geological Survey (i.e. total precipitation during the driest quarter, annual mean temperature, maximum temperature during the warmest month, mean temperature of the warmest quarter and mean temperature of the coldest quarter) [63]. Model optimization was performed by tuning the *mtry* parameter (number of variables randomly sampled as candidates at each split) across three values.

Performance was quantitatively assessed via confusion matrix analysis, which provided classification accuracy metrics and Cohen's Kappa statistics. The optimal *mtry* value was determined based on cross-validation results, with overall accuracy and Kappa values recorded as performance indicators.

For spatial prediction classification, a custom function (*get_class_none*) to the probability outputs was applied. This function assigns class membership based on the maximum predicted probability. To account for classification uncertainty, observations with maximum probabilities below 0.6 were designated as "non-classified", while observations exceeding this threshold were assigned to their respective highest probability class (clusters 1, 2 or 3). The spatial prediction was implemented using the *calc* function, and the resulting classification was visualized through raster mapping using the *raster* R package (v3.6-32) [64].

### Statistical analysis

Statistical differences were assessed using various tests depending on the comparison. Differences between sponge sample types, feces species origin, and between fecal and sponge samples were evaluated using Fisher’s exact test and chi-square test, implemented in R software version 4.4.1. Additionally, the Wilcoxon Mann–Whitney test was used for PCR CT-values comparisons, performed using the *dplyr* (v1.1.4) and *rstatix* (v0.7.2) R packages [56, 65].

For the variables used in the HCPC analysis, statistical differences between clusters were also assessed using the Kruskal–Wallis and Wilcoxon Mann–Whitney tests, again utilizing the *dplyr* and *rstatix* R packages [56, 65].

## Results

Our findings revealed that methodology, particularly sampling design, influences the efficacy of ENAD for non-invasive monitoring in natural areas. A clear influence of environmental factors on pathogen-related marker detection on natural surfaces was found, underscoring the importance of optimized sampling protocols for reliable environmental surveillance.

### Surface sponges ENAD

**Figure 1:**
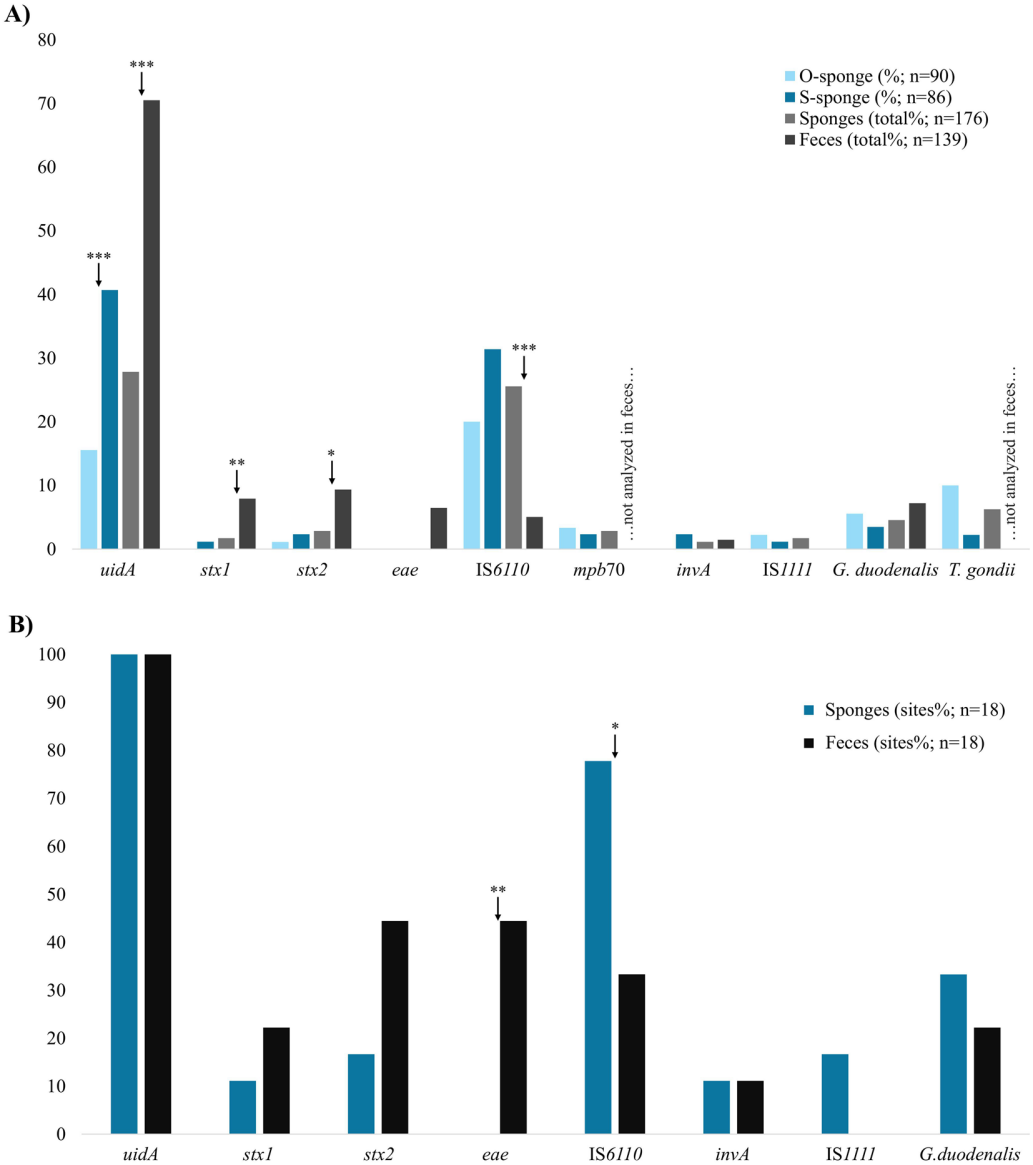
**Comparison of the proportion of total positive samples between O-sponges and S-sponges (A), as well as the proportion of positive sites between feces and sponges** (**B**). Pathogen-related markers: *Escherichia coli* (*uidA*, *stx1*, *stx2* and *eae*), *Mycobacterium tuberculosis* complex (IS*6110* and *mpb*70), *Salmonella* spp. (*invA*), *Coxiella burnetii* (IS*1111*), *Giardia duodenalis*, and *Toxoplasma gondii*. Statistical significance of the Fisher’s exact test and chi-square test are indicated as follows: *p* < 0.05 (*), *p* < 0.01 (**), and *p* < 0.001 (***).

Surface sampling with sponges showed capability to detect pathogen-related markers (*E. coli,* MTC, *Salmonella* spp., *C. burnetii*, *G. duodenalis* and *T. gondii*) in natural areas. However, methodology in sponge sampling (O-sponges or S-sponges) influenced specific marker detectability. In this study, S-sponges showed higher utility for detecting *E. coli* markers, since a significantly higher proportion of S-sponges tested positive for the *uidA* gene (40.69%) compared to the O-sponges (15.55%) (Figure 1A). Regarding other *E. coli* markers, *stx1* was detected only in S-sponges and *stx2* tended to be detected more frequently in S-sponges. The PCR CT-values also tended to be lower in S-sponges for the three *E. coli* markers (*p* = 0.01 for *uidA* CT-values; Additional file 1). For the MTC markers IS*6110* and *mpb*70, no significant differences in proportion of positives were found between O-sponges and S-sponges, however, for IS*6110* the PCR CT-values were significantly lower in the S-sponges (*p* = 0.01; Additional file 1). Regarding other bacteria, the *Salmonella* spp. marker *invA* was detected only in S-sponges, while no differences were found between sponge samples for the *C. burnetii* marker IS*1111*, despite PCR CT-values tended to be lower in S-sponges (Additional file 1). Parasite ENAD showed no significant differences between sponge samples, but a trend to a higher detectability of *G. duodenalis*. and *T. gondii* in the O-sponges was observed (Additional file 1).

The highest proportion of positive sampling points was found for the *uidA* marker (48.80%), detected in 100% of the study sites, followed by the MTC marker IS*6110* (42.22% of sampling points) detected in 77.77% of the locations. The parasites were the second group of markers with more positive sampling points (12.22% for *T. gondii* and 8.89% for *G. duodenalis*) and study sites presence (50% for *T. gondii* and 33.30% for *G. duodenalis*). Other markers showed a proportion of positive sampling points ranging from 2.22 to 5.55% and study sites from 11.11 to 16.66% (Additional files 1 and 2).

### Fecal ENAD

Several molecular markers associated with bacteria and parasites were detected in wild boar and wild ruminant fecal samples. These included *E. coli* markers, MTC marker IS*6110*, *Salmonella* spp. marker *invA*, *G. duodenalis*, *Blastocysitis* sp., *B. coli*, *E. bieneusi* and *Encephalitozoon cuniculi*. From a general perspective, no apparent differences were found between wild boar feces and ruminant feces, except for specific markers.

The *E. coli* markers showed no apparent differences between wild boar and wild ruminant feces. However, a trend to higher proportion of positives for *uidA*, *stx1*, *stx2* and *eae* markers in wild boar feces was found (Additional file 1). Regarding the PCR CT-values, significant differences were found for *uidA* (*p* = 0.003, with lower values in wild boar feces) and *stx1* (*p* = 0.03, with lower values in wild ruminant feces). The MTC marker IS*6110* was detected both in wild boar and wild ruminant feces with no significant differences for positivity and CT-values. The *Salmonella* marker *invA* was found in 4.34% of the wild boar feces (Additional file 1). Parasite markers showed no difference between ruminant and wild boar feces. *G. duodenalis*, *Blastocysits* sp., and *Enc. cuniculi* were found in both fecal samples, while *B. coli* and *E. bieneusi* were found only in wild boar feces (Additional file 1).

The highest proportion of positive study sites in feces samples were for *E. coli* markers (*uidA*-100%-, *stx2*-44.44%-, *eae*-44.44%- and *stx1*-22.22%) followed by MTC marker IS*6110* (33.33%) and three parasite markers (*G. duodenalis*-22.22%-, *Blastocystis* sp.-22.22%- and *Enc. cuniculi*-16.66%-). Finally, *B. coli* and *invA* were positive in 11.11% and *E. bieneusi* in 5.56% of the study sites (Additional file 2).

### Comparing surface sponges and fecal ENAD

Among the pathogen-related markers analyzed in both sponge and fecal samples, significant differences were found in opposite senses for *E. coli* and MTC markers. All *E. coli* markers were more positive in fecal samples (*uidA p* < 0.001; *stx1 p* = 0.02; *stx2 p* = 0.03; *eae* was detected only in fecal samples; Figure 1A). Consistently, the PCR CTs for *uidA* were lower in fecal samples (*p* = 0.01; Additional file 1). By contrast, the IS*6110* was detected in 25.57% of sponge samples, while only 5.04% of fecal samples tested positive (*p* < 0.001) (Figure 1A). The IS*6110* CT-values were also lower in sponge samples (*p* = 0.005; Additional file 1). The MTC marker *mpb*70 was also detected in sponge samples. Other bacteria like *C. burnetii* (IS*1111*) were detected only in sponge samples, too (Additional file 1). Regarding parasites, there were no significant differences in *Giardia* detectability between surface or fecal ENAD, however, a higher positivity trend in feces was observed (Figure 1A; Additional file 1). The CT-values for *G. duodenalis* were significantly lower in fecal samples (*p* = 0.004).

A comparison of the proportion of sites positive to pathogen-related markers between sponge and fecal samples is shown in Figure 1B. Significant differences were found in the number of positive study sites for *eae* and IS*6110* markers (*p* = 0.003 and *p* = 0.02, respectively; Additional file 2).

### Factors driving ENAD of pathogen-related markers in sponge samples

Molecular marker detection in sponge samples was strongly associated with climatic variables (latitude influence) rather than biological factors typically linked to pathogen circulation. Notably, pathogen-related ENAD showed a negative relationship with serological sanitary data and wild ungulate abundance and a positive relationship with grazing ruminant livestock (see “Correlations with pathogen-related markers diversity” section and cluster description). The subsequent analysis examines the specific factors influencing ENAD of pathogen-related markers on surfaces.

A study site clustering was performed to assess how land cover, climate, mammal community and health variables influence pathogen-related marker detection (Additional file 4). Three groups (clusters) of locations were established, seven locations belonging to cluster 1, six to cluster 2 and five to cluster 1 (Additional file 3). Clusters were mainly differentiated by the influence of latitude. Cluster 1, the southern one and representing Mediterranean climate, presents higher temperatures and lower precipitation rates than cluster 2 with intermediate climatic characteristics and cluster 3, with lower temperatures and higher precipitation rates, typical of Atlantic climate (Additional file 4). Despite no significant differences when comparing the three clusters, a positive trend from southern locations to northern locations was seen for forest, grassland and urban land use (Additional file 4). Regarding mammal communities, the relative weight of red deer in the mammal community was significantly higher in cluster 1, while carnivores had significantly more weight in clusters 2 and 3. Ruminant livestock and wild boar tended to have more weight in cluster 3. Co-exposure rates showed significant differences between clusters, being higher in cluster 1 and lower in cluster 3 (Additional file 4).

The diversity of pathogen-related markers (H’) detected in sponges (ENAD) showed a non-significant trend among the three clusters (*p* = 0.11; Additional file 4), with highest values in cluster 3 (1.26 ± 0.23), followed by cluster 2 (1.13 ± 0.31) and cluster 1 (0.77 ± 0.47). However, pairwise comparisons revealed significant differences between cluster 1 and cluster 3 (Wilcoxon Mann–Whitney test: W = 5; *p* = 0.04). The comparisons between clusters 2 and 3 (Wilcoxon Mann–Whitney test: W = 12; *p* = 0.66) and clusters 1 and 2 (Wilcoxon Mann–Whitney test: W = 11; *p* = 0.18) showed no significant differences. Pathogen-related marker richness in sponge samples followed the same pattern, with highest values in cluster 3 (range: 3–6; mean: 4.20 ± 1.30), followed by cluster 2 (range: 2–5; mean: 3.50 ± 1.05) and cluster 1 (range: 1–5; mean: 2.57 ± 1.27; *p* = 0.09; Additional file 4).

#### Correlations with pathogen-related marker diversity

Molecular marker diversity detected on surfaces showed marginally significant positive correlations with forest cover (r^2^ = 0.42; *p* = 0.08) and grassland (r^2^ = 0.41; *p* = 0.09). Latitude demonstrated a strong positive correlation with marker diversity (r^2^ = 0.65; *p* = 0.003). For the climatic variables, precipitation during the driest quarter was positively correlated with pathogen-related marker diversity (r^2^ = 0.57; *p* = 0.01), while temperature variables showed significant negative correlations, including annual mean temperature (r^2^ = −0.71; *p* < 0.001), maximum temperature during the warmest month (r^2^ = −0.66; *p* = 0.003), mean temperature of the warmest quarter (r^2^ = −0.74; *p* < 0.001), and mean temperature of the coldest quarter (r^2^ = −0.59; *p* = 0.01). Additionally, pathogen-related marker diversity showed a significant positive correlation with relative weight of ruminant livestock in the mammal community (r^2^ = 0.62; *p* = 0.006) and a negative correlation with co-exposure rates (r^2^ = −0.49; *p* = 0.04). Non-significant results were established for carnivore, red deer and wild boar relative weights in the community (r^2^ = 0.36 *p* = 0.14; r^2^ = −0.28 *p* = 0.26; r^2^ = −0.15 *p* = 0.55, respectively).

#### Distribution of individual pathogen-related markers among clusters

Cluster 3 locations had higher proportion of positive study sites to all analyzed molecular markers in surface eDNA (ranging from 20 to 100% of cluster 3 locations), except for *T. gondii* with higher positivity in cluster 2 locations (Table 2). The *E. coli* marker *uidA* was detected in all study sites, while MTC marker *mpb*70 and *Salmonella* spp. marker *invA* were not detected in cluster 1 locations. Similarly, *E. coli* marker *stx1* and *Salmonella* spp. marker *invA* were also not detected in cluster 2.

**Table 2:** **Proportion (%) of positive communities in each cluster per pathogen marker analyzed**

Pathogen marker	Cluster 1 (% locations)	Cluster 2 (% locations)	Cluster 3 (% locations)
IS*6110* MTC	71.43	66.67	100
*mpb*70 MTC	0	16.67	40
*uidA Escherichia coli*	100	100	100
*stx1 Escherichia coli*	14.29	0	20
*stx2 Escherichia coli*	14.29	16.67	20
*invA Salmonella* spp.	0	0	40
IS*1111 Coxiella burnetii*	14.29	16.67	20
*Giardia duodenalis*	28.57	33.33	40
*Toxoplasma gondii*	14.29	100	40

*MTC Mycobacterium tuberculosis* complex.

#### Spatial distribution of de clusters in the Iberian Peninsula

**Figure 2:**
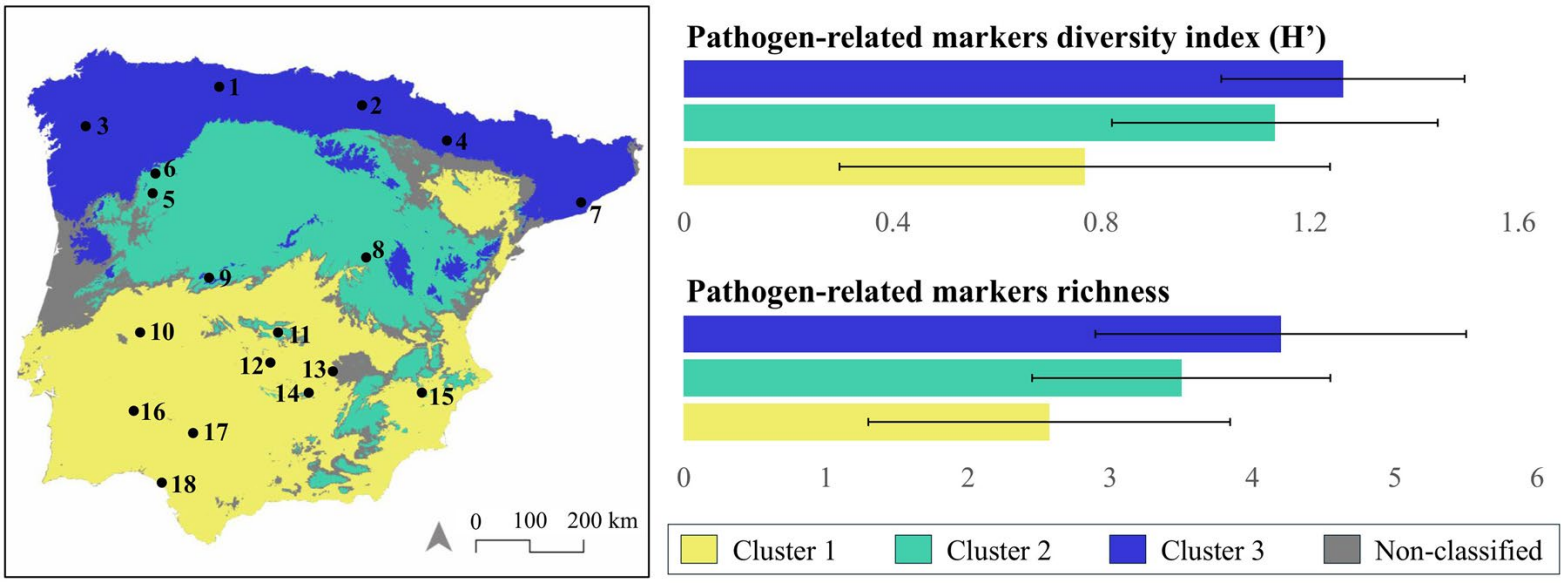
**Spatial distributions of the clusters in the Iberian Peninsula with varying pathogen-related markers diversity index (H’) and richness** (Additional files 4, 7). “Non-classified” indicates areas with classification probabilities below 0.6 for each cluster (see “Spatial distribution modeling of clusters in the Iberian Peninsula” from Material and Methods section). In the bar chart is represented the mean and the standard deviation of markers diversity and richness per cluster. Geographic location of the study sites: (1) Cantabrian Mountains; (2) Llanada alavesa valley; (3) Northern coast; (4) Pre-Pyrenees; (5) Trás-os-montes region; (6) Trás-os-montes region; (7) Catalonia coast (8) Iberian System; (9) Central System; (10) San Pedro Mountains; (11) Toledo Mountains; (12) Guadiana Valley; (13) Campo de Montiel region; (14) Sierra Morena Mountains; (15) Cordillera Bética Mountains; (16) Baixo Alentejo region; (17) North Seville Mountains; (18) Guadalquivir Valley.

Based on the factors driving molecular detection of pathogen-related markers in sponge samples, a cluster distribution map was created using land cover and climatic data from open data sources. This map distributes the three defined clusters across the Iberian Peninsula, each one characterized by different environmental conditions that influence ENAD. The confusion matrix of the predicted clustering map demonstrates balanced performance metrics, with an AUC, F1 score, precision, and recall all measuring 0.89. This indicates that errors are evenly distributed across classes, with equal rates of false positives and false negatives for each class (Additional files 5 and 6). This map (Figure 2 and Additional file 7) provides an initial reference for expected pathogen-related marker diversity and richness in sponge samples across the Iberian Peninsula.

## Discussion

This study identifies the determinants that modulate environmental nucleic acid detection (ENAD) using the detection of pathogen-related environmental DNA (eDNA) markers as a proof of concept. Our findings provide relevant guidelines concerning the collection of fecal samples and the use of sponges for surface sampling, thus facilitating methodological decision-making and improved sampling designs. Through a comparative analysis of diverse episystems, we show that climatic variables, in partial conjunction with land use patterns, constitute the predominant factors that drive pathogen-related marker detection on surfaces in natural ecosystems.

The detection of different molecular markers in sponge samples taken from surfaces revealed the potential of this non-invasive methodology for studying the presence of pathogens in natural areas. When differentiating between the two types of sponge samples (O-sponge and S-sponge), significant differences were observed. The detection of the markers *uidA*, *stx1*, *stx2* (*E. coli*), IS*6110* (MTC), and *invA* (*Salmonella* spp.) was consistently higher in S-sponges, which are expected to have higher fecal contamination rates. Conversely, the detection of *mpb*70 (MTC), IS*1111* (*C. burnetii*), *G. duodenalis*, and *T. gondii* was slightly higher in O-sponges. This result suggests that it might be a good strategy to combine O- and S-sponge samples to cover the full range of detectable pathogen-related markers.

When comparing the results of sponge samples from this study, collected in natural areas, with those reported by Herrero-García et al. [14] from hoofstock farm premises, as shown in Table 3, differences can be observed. Sample positivity was always higher on farm premises than in natural habitats except for IS*6110*. This difference could be explained by the existence of livestock sanitation campaigns for livestock and by the possible exclusion of other wild ungulates by livestock [66, 67]. However, it is relevant to note that all markers detected on farm environments were also detected in natural areas.

**Table 3:** **Comparison of PCR-CT values and % of positive sponge samples between previous studies done in farms by Herrero-García et al. **[14]** and this study**

Pathogen marker	Herrero-García et al. [14]				This study			
	% of positive samples	PCR-CT values			% of positive samples	PCR-CT values		
		Mean	Max	Min		Mean	Max	Min
IS*6110*	17.73	39.08	43.90	36.04	25	37.14	39.97	31.57
IS*1111*	5.88	34.19	37.80	31.61	1.67	37.80	39.51	35.35
*uidA*	81.56	35.19	43.84	23.93	27.22	35.92	39.41	26.20
*stx1*	2.13	41.24	43.28	40.03	1.67	35.89	38.87	33.38
*stx2*	2.84	35.50	37.94	33.36	2.78	32.41	35.16	30.09
*eae*	2.84	30.29	32.33	28.87	0.00	0.00	0.00	0.00
*invA*	8.51	38.42	41.58	33.61	1.11	36.17	36.29	36.05

Max. and Min. refers to maximum and minimum CT-values per each marker.

In natural environments, significantly higher positivity rates for molecular markers were obtained in feces collected from the environment compared to sponges. However, this difference appears to be driven by the greater detection of markers associated with *E. coli* (*uidA*, *stx1*, *stx2*, and *eae*), which presumably could be more easily found in fecal samples than in surface samples in natural areas.

Thus, given that most markers detected in feces can also be detected in surface sponges, the latter seems to represent the optimal choice.

One of the main limitations to implementing ENAD in health and ecological surveillance programs is the lack of information on how different variables, such as climatic factors, affect DNA/RNA detection performance in non-invasive samples [7]. In this study, we showed that diversity and richness of pathogen-related markers detected in sponges was mainly driven by climatic factors. This might explain why counterintuitively, pathogen-related ENAD showed a negative relationship with serological indicators of pathogen exposure. This does not fit with established knowledge regarding vertebrate community influences on pathogen dynamics and host health [24].

In this study, we observed that in areas at higher latitudes, which have higher precipitation rates and lower temperatures, the detection of pathogen-related markers on surface samples was higher. We found the greatest diversity of markers in sponges from cluster 3, which is also the cluster with the highest precipitation rates, lower/moderate temperatures, and greater forest and grassland cover. All these factors might contribute to a higher proportion of shaded areas, higher humidity, and less extreme temperatures. These conditions are important for the survival of certain pathogens and, consequently, for the persistence of their nucleic acids. For example, Fine et al. [68] found that *Mycobacterium bovis* has a lower survival rate in the environment during spring/summer, when temperatures and UV light incidence are highest. These authors also reported that shade influences the survival times of this pathogen. This is consistent with our findings, which reflect a marginally significant positive effect of forest and grassland cover on the diversity of molecular markers detected in sponge samples. Our results also demonstrate that the diversity of molecular markers detected correlates positively with precipitation indexes and negatively with temperature. Similarly, the findings of Williams et al. [69] showed that *E. coli* O157 has higher survival rates in more humid environments and at lower temperatures [69]. It is important to emphasize that the previously referenced studies address the survival of the microorganism itself, whereas in the present study, only bacterial DNA may be detected, reflecting eDNA persistence rather than viable microorganism load [70]. The results could indicate that environmental conditions are likely to influence the preservation of microbial DNA in the environment.

It is noteworthy that while no significant differences were found in the diversity index of markers detected across different clusters, significant differences emerged when pairwise comparisons were made between cluster 1 and cluster 3. From this, it can be inferred that cluster 2 represents a transitional environment with relatively intermediate climatic and habitat characteristics, since the pairwise comparison of this cluster with the others does not have significant differences.

The balanced distribution of errors between false positives and false negatives indicates that the model does not systematically favor any specific cluster, reinforcing the reliability of our predictions for different geographic regions of the Iberian Peninsula. The generated predictive map represents a valuable tool for guiding sampling strategies in future studies. Identifying areas with a higher probability of detecting genetic material from microorganisms will optimize sampling resources and efforts. However, it is important to consider that this model constitutes a first approximation that will need to be refined by incorporating additional variables and increasing the sample size, among other considerations. Altogether, we identify habitat and climate characteristics as the main driver of pathogen-related marker detection in studies at larger geographic scales.

This study has several limitations. First, although pathogen-related markers were proposed as a study model for assessing factors influencing ENAD, analyses at limited and smaller spatial scales are needed to evaluate drivers of potential pathogen contamination in the environment, such as mammal community characteristics, among others. Given the limited number of study sites, our findings should be interpreted as a generalization of the factors influencing ENAD within Iberian-like environments. The lack of standardized surface area sampled with sponges represents an inherent challenge in field-based eDNA monitoring, since DNA is expected to be distributed unevenly across different substrates, hindering strict standardization. Our approach addressed this by sampling multiple surface types within a defined area (50 m^2^), thereby capturing environmental variability while maintaining spatial consistency across sites. Additionally, some markers were analyzed only in fecal or sponge samples due to limitations in the remaining sample volumes. Furthermore, the detectability of fecal samples from different species is determined by multiple factors, such as animal density and study site characteristics including type of vegetation and topography. There is also a limitation, especially in the wild ruminant group, in correctly differentiating feces of one species from another. Finally, a clear limitation for molecular analysis and the standardization of results on fecal samples is its variable conservation status.

Overall, our results suggest that combining O- and S-sponge samples may be an effective strategy to maximize the detection of pathogen-related markers. However, since most markers identified in fecal samples were also detectable in surface sponges, surface sponges seem to be the most practical and efficient option. Furthermore, our findings highlight habitat and climate characteristics as key drivers of ENAD at broader geographic scales.

## Supplementary Information

Additional file 1: **Comparison of positivity and PCR CT-values for sponge sample types, feces origin and overall sponges and fecal samples, per molecular marker, sampling point and study site. This file provides a comparison of positivity and PCR CT-values for sponge sample types, feces origin and overall sponges and fecal samples, per molecular marker, sampling point and study site.**

Additional file 2: **Proportion of positive study sites (*n*** **= 18) per pathogen-related marker. This table compiles information related to the proportion of positive study sites (*n*** **= 18) per pathogen-related marker.**

Additional file 3: **Factor map showing the proportion (%) of variance explained by both HCPC dimensions (A), and hierarchical clustering dendrogram (B). This figure represents the factor map showing the proportion of variance explained by both HCPC dimensions, and hierarchical clustering dendrogram.**

Additional file 4: **Clustering variables differences among established clusters. This file provides numerical information regarding the clustering variables differences among established clusters.**

Additional file 5: **Confusion matrix and statistics. This file contains the confusion matrix and statistics of predictive analyses.**

Additional file 6: **Prediction of the LOOCV using random forest method. This table show the accuracy of the predictive analyses across the different clusters.**

Additional file 7: **Probability of each map pixel belonging to a cluster. This figure represents the probabilities of each map pixel belonging to a cluster.**

Additional file 8: **Methods. This file describes in detail the PCR protocols and mammal community parameters.**

## Data Availability

The datasets generated and/or analysed during the current study are available in the Zenodo repository: 10.5281/zenodo.17099159. Further supplementary information and data are available are available from the corresponding author on reasonable request.
