# Supplementary material for: Tracking pathogen-related markers with eDNA in natural areas: how environmental factors shape surveillance strategies
Source: Vet Res. 2026 Apr 28;57:90. doi: 10.1186/s13567-026-01746-6 (PMC13214320; doi:10.1186/s13567-026-01746-6)
Supplement: Supplementary file 1 — Additional file 1: Comparison of positivity and PCR CT-values for sponge sample types, feces origin and overall sponges and fecal samples, per molecular marker, sampling point and study site. This file provides a comparison of positivity and PCR CT-values for sponge sample types, feces origin and overall sponges and fecal samples, per molecular marker, sampling point and study site. [file 13567_2026_1746_MOESM1_ESM.docx]

**Supplementary table 1**. Comparison of positivity and PCR CT-values for sponge sample types, feces origin and overall sponges and fecal samples, per molecular marker, sampling point and study site.

| **Pathogen-related marker** | **Value** | **O-sponge (n=90)** | **S-sponge (n=86)** | **STATISTICS RESULTS** | **Sampling points (sponges)** | **Wild boar feces (n=46)** | **Wild ruminant feces (n=93)** | **STATISTICAL RESULTS** | **Sponges (total; n=176)** | **Feces (total; n=139)** | **STATISTICAL RESULTS** |
| --- | --- | --- | --- | --- | --- | --- | --- | --- | --- | --- | --- |
| ***E. coli* (*uidA*)** | **Positivity (%)** | 15.55 | 40.69 | χ²=12.61; *p*=0.0004 | 48.8 | 78.26 | 66.66 | χ²=1.47; *p*=0.23 | 27.84 | 70.5 | χ²=55.09; *p*=1.15e^-13^ |
|  | **PCR-CT (x̄±SD)** | 37.28  ±2.32 | 35.37  ±2.92 | W=359.5; *p*=0.01 |  | 32.39  ±4.23 | 34.98  ±3.79 | W=711.5; *p*=0.003 | 35.92  ±2.87 | 34.03  ±4.14 | W=1800; *p*=0.01 |
| ***E. coli* (*stx1*)** | **Positivity (%)** | 0 | 1.16 | na | 3.3 | 13.04 | 6.45 | OR=0.38; *p*=0.18 | 1.70 | 7.91 | χ²=5.66; *p*=0.02 |
|  | **PCR-CT (x̄±SD)** |  | 35.88  ±2.77 | na |  | 37.46  ±4.09 | 31.87  ±3.87 | W=27; *p*=0.03 | 35.88  ±2.77 | 34.92  ±4.79 | W=13; *p*=0.66 |
| ***E. coli* (*stx2*)** | **Positivity (%)** | 1.11 | 2.32 | OR=4.31; *p*=0.2 | 5.55 | 13.04 | 7.52 | OR=0.55; *p*=0.36 | 2.84 | 9.35 | χ²=4.96; *p*=0.03 |
|  | **PCR-CT (x̄±SD)** | 35.16 | 31.71  ±1.47 | na |  | 34.87  ±2.48 | 33.48  ±2.30 | W=24; *p*=0.73 | 32.41  ±1.99 | 34.13  ±2.30 | W=47; *p*=0.17 |
| ***E. coli* (*eae*)** | **Positivity (%)** | 0 | 0 | na | 0 | 13.04 | 3.22 | OR=0.22; *p*=0.05 | 0 | 6.47 | na |
|  | **PCR-CT (x̄±SD)** | na | na | na |  | 31.66  ±4.40 | 31.95  ±0.64 | W=6; *p*=0.55 | na | 31.38  ±3.34 | na |
| **MTC (IS*6110*)** | **Positivity (%)** | 20 | 31.39 | χ²=2.43; *p*=0.12 | 42.22 | 6.52 | 4.30 | OR=0.65; p=0.68 | 25.57 | 5.04 | χ²=22.29; p=2.35e-06 |
|  | **PCR-CT (x̄±SD)** | 38.02  ±1.47 | 36.54  ±1.99 | W=353; *p*=0.01 |  | 38.65  ±0.94 | 40.29  ±1.87 | W=3; p=0.40 | 37.14  ±1.93 | 39.58  ±1.67 | W=262.5; p=0.01 |
| **MTC (*mpb*70)** | **Positivity (%)** | 3.33 | 2.32 | OR=0.69; *p*=1 | 4.44 | na | na | na | 2.84 | na | na |
|  | **PCR-CT (x̄±SD)** | 38.84  ±0.67 | 37.48  ±2.89 | W=4;  *p*=0.80 |  | na | na | na | 38.30  ±1.69 | na | na |
| ***Salmonella* spp. (*invA*)** | **Positivity (%)** | 0 | 2.32 | na | 2.22 | 4.34 | 0 | na | 1.14 | 1.44 | OR=0.79; *p*=1 |
|  | **PCR-CT (x̄±SD)** | na | 36.17  ±0.17 | na |  | 37.43  ±0.67 | na | na | 36.17  ±0.17 | 37.43  ±0.67 | W=4; *p*=0.33 |
| **C. burnetii**  **(IS*1111*)** | **Positivity (%)** | 2.22 | 1.16 | OR=0.52; *p*=1 | 3.33 | 0 | 0 | na | 1.7 | 0 | na |
|  | **PCR-CT (x̄±SD)** | 39.03  ±0.68 | 35.35 | na |  | na | na | na | 37.80  ±2.18 | na | na |
| ***G. duodenalis*** | **Positivity (%)** | 5.55 | 3.48 | OR=0.61; *p*=0.72 | 8.89 | 6.52 | 7.53 | OR=1.16; *p*=1 | 4.55 | 7.19 | χ²=0.59; *p*=0.44 |
|  | **PCR-CT (x̄±SD)** | 38.06  ±1.35 | 38.20  ±1.14 | W=7;  *p*=1 |  | 32.60  ±5.64 | 35.90  ±2.85 | W=6; *p*=0.36 | 38.11  ±1.19 | 34.92  ±3.88 | W=16.5; *p*=0.004 |
| ***T. gondii*** | **Positivity (%)** | 10 | 2.22 | OR=0.21; *p*=0.05 | 12.22 | na | na | na | 6.25 | na | na |
| ***M. a. paratuberculosis* (IS*900*)** | **Positivity (%)** | na | na | na | na | 0 | 0 | na | na | 0 | na |
| ***Blastocystis* sp.** | **Positivity (%)** | na | na | na | na | 6.52 | 4.3 | OR=0.65; *p*=0.68 | na | 5.04 | na |
| ***B.coli*** | **Positivity (%)** | na | na | na | na | 8.69 | 0 | na | na | 2.88 | na |
| ***E.bieneusi*** | **Positivity (%)** | na | na | na | na | 2.17 | 0 | na | na | 0.72 | na |
|  | **PCR-CT (x̄±SD)** | na | na | na |  | 35 | na | na | na | 35 | na |
| ***E.cuniculi*** | **Positivity (%)** | na | na | na | na | 2.17 | 2.15 | OR=0.99; *p*=1 | na | 2.16 | na |
|  | **PCR-CT (x̄±SD)** | na | na | na |  | 36 | 37.60  ±0.28 | na | na | 37.07  ±0.95 | na |
| ***Brucella* spp. (IS*711*)** | **Positivity (%)** | na | na | na | na | 0 | 0 | na | na | 0 | na |
| ***Cryptosporidium* spp.** | **Positivity (%)** | 0 | 0 | na | 0 | 0 | 0 | na | 0 | 0 | na |

—“OR”=odds ratio; “na”=no available; “χ²”=chi-square value; “SD”=standard deviation; “W”=Wilcoxon test parameter— *Escherichia coli* (*uidA*, *stx1*, *stx2* and *eae*), *Mycobacterium tuberculosis* complex -MTC- (IS*6110* and *mpb70*), *Salmonella* spp. (*invA*), *Coxiella burnetii* (IS*1111*), *Brucella* spp. (IS*711*), *M. avium* subp. *paratuberculosis* (IS*900*), *Balantioides coli, Blastocystis sp.*, *Cryptosporidium* spp, *Encephalitozoon* spp, *Enterocytozoon bieneusi, Giardia* *duodenalis*, and *Toxoplasma gondii*.
