# Supplementary material for: Tracking pathogen-related markers with eDNA in natural areas: how environmental factors shape surveillance strategies
Source: Vet Res. 2026 Apr 28;57:90. doi: 10.1186/s13567-026-01746-6 (PMC13214320; doi:10.1186/s13567-026-01746-6)
Supplement: Supplementary file 2 — Additional file 2: Proportion of positive study sites (n = 18) per pathogen-related marker. This table compiles information related to the proportion of positive study sites (n = 18) per pathogen-related marker. [file 13567_2026_1746_MOESM2_ESM.docx]

**Supplementary table 2.** Proportion of positive study sites (n=18) per pathogen-related marker.

| **Pathogen-related marker** | **Sponges (sites %)** | **Feces (sites %)** | **STATISTICAL RESULTS** |
| --- | --- | --- | --- |
| *E. coli* (*uidA*) | 100 | 100 | χ²=0; *p*=1 |
| *E. coli* (*stx1*) | 11.11 | 22.22 | OR=0.17; *p*=0.06 |
| *E. coli* (*stx2*) | 16.67 | 44.44 | OR=0.26; *p*=0.15 |
| *E. coli* (*eae*) | 0 | 44.44 | OR=0; *p*=0.003 |
| MTC (IS*6110*) | 77.77 | 33.33 | χ²=5.5; *p*=0.02 |
| *Salmonella* spp. (*invA*) | 11.11 | 11.11 | OR=1; *p*=1 |
| *C. burnetii* (IS*1111*) | 16.66 | 0 | OR=Inf; *p*=0.23 |
| *G. duodenalis* | 33.30 | 22.22 | χ²=0.12; *p*=0.73 |
| *T. gondii* | 50 | na | na |
| *M. a. paratuberculosis* (IS*900*) | na | 0 | na |
| *Blastocystis* sp. | na | 22.22 | na |
| *B.coli* | na | 11.11 | na |
| *E.bieneusi* | na | 5.56 | na |
| *E.cuniculi* | na | 16.66 | na |
| *Brucella* spp. (IS*711*) | na | 0 | na |
| *C.parvum* | 0 | 0 | na |
| MTC (*mpb*70) | 16.66 | na | na |

—“OR”=odds ratio; “na”=no available; “χ²”=chi-square value— *Escherichia coli* (*uidA*, *stx1*, *stx2* and *eae*), *Mycobacterium tuberculosis* complex -MTC- (IS*6110* and *mpb70*), *Salmonella* spp. (*invA*), *Coxiella burnetii* (IS*1111*), *Brucella* spp. (IS*711*), *M. avium* subp. *paratuberculosis* (IS*900*), *Balantioides coli, Blastocystis sp.*, *Cryptosporidium* spp, *Encephalitozoon* spp, *Enterocytozoon bieneusi, Giardia* *duodenalis*, and *Toxoplasma gondii*.
