# Supplementary material for: Tracking pathogen-related markers with eDNA in natural areas: how environmental factors shape surveillance strategies
Source: Vet Res. 2026 Apr 28;57:90. doi: 10.1186/s13567-026-01746-6 (PMC13214320; doi:10.1186/s13567-026-01746-6)
Supplement: Supplementary file 3 — Additional file 3: Factor map showing the proportion (%) of variance explained by both HCPC dimensions (A), and hierarchical clustering dendrogram (B). This figure represents the factor map showing the proportion of variance explained by both HCPC dimensions, and hierarchical clustering dendrogram. [file 13567_2026_1746_MOESM3_ESM.docx]

**
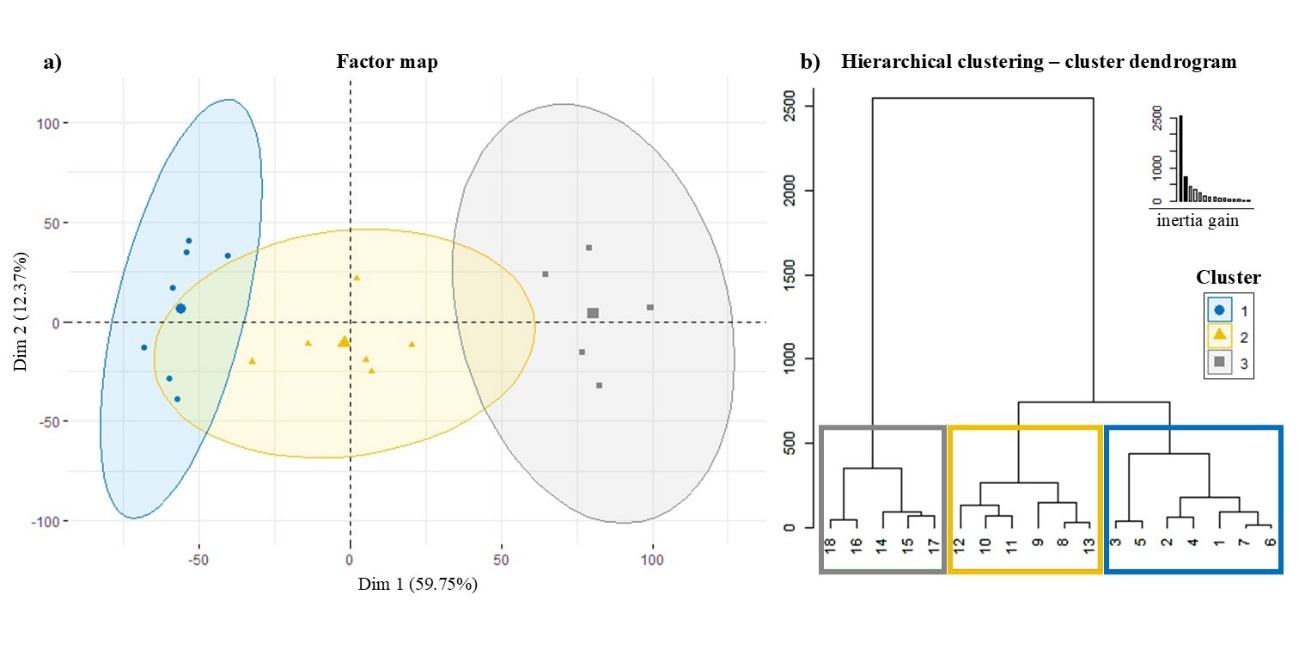
**

**Supplementary figure 1.** Factor map showing the proportion (%) of variance explained by both HCPC dimensions (a), and hierarchical clustering dendrogram (b).
