# Supplementary material for: Tracking pathogen-related markers with eDNA in natural areas: how environmental factors shape surveillance strategies
Source: Vet Res. 2026 Apr 28;57:90. doi: 10.1186/s13567-026-01746-6 (PMC13214320; doi:10.1186/s13567-026-01746-6)
Supplement: Supplementary file 4 — Additional file 4: Clustering variables differences among established clusters. This file provides numerical information regarding the clustering variables differences among established clusters. [file 13567_2026_1746_MOESM4_ESM.docx]

**Supplementary table 3.** Clustering variables differences among established clusters.

| **VARIABLE GROUP** | **VARIABLE** | **CLUSTER 1 (x̄±SD)** | **CLUSTER 2 (x̄±SD)** | **CLUSTER 3 (x̄±SD)** | **χ²-value** | ***p*-value** |
| --- | --- | --- | --- | --- | --- | --- |
| **Land cover** | **Urban use (%)** | 0.02±0.06 | 0.14±0.23 | 0.53±0.75 | 3.11 | 0.21 |
|  | **Forest coverage (%)** | 34.31±30.26 | 32.23±22.72 | 55.36±19.79 | 2.39 | 0.30 |
|  | **Grassland coverage (%)** | 0.90±2.39 | 1.01±1.39 | 3.44±4.57 | 2.41 | 0.30 |
|  | **Bare land (%)** | 0.90±2.38 | 2.48±6.06 | 1.49±2.79 | 1.85 | 0.40 |
|  | **Shrubland coverage (%)** | 30.03±30.01 | 20.85±24.40 | 22.41±10.53 | 0.31 | 0.86 |
| **Climate** | **Precipitation during the driest quarter (mm)** | 32.43±8.06 | 66.50±14.92 | 145.60±21.73 | 14.62 | 0.001 |
|  | **Maximum temperature during the warmest month (°C)** | 32.75±1.07 | 29.50±3.40 | 25.38±2.82 | 10.40 | 0.006 |
|  | **Annual mean temperature (°C)** | 15.55±1.45 | 11.92±1.46 | 11.64±2.31 | 9.76 | 0.008 |
|  | **Mean temperatures during the warmest quarter (°C)** | 23.86±0.51 | 20.48±2.07 | 18.63±2.66 | 10.36 | 0.006 |
|  | **Mean temperatures during the coldest quarter (°C)** | 8.32±2.24 | 4.65±1.35 | 5.53±2.16 | 8 | 0.02 |
| **Latitude** | | 38.45±0.88°N | 40.32±1.49°N | 42.50±0.67°N | 11.51 | 0.003 |
| **Mammal community** | **Red deer relative weight (%)** | 61.72±16.31 | 16.55±20.07 | 6.45±14.35 | 11.62 | 0.003 |
|  | **Carnivore community relative weight (%)** | 7.63±4.52 | 20.11±13.18 | 20.90±12.46 | 8.73 | 0.01 |
|  | **Grazing domestic ruminants relative weight (%)** | 1.20±2.88 | 1.61±1.36 | 3.27±2.99 | 5.24 | 0.07 |
|  | **Wild boar relative weight (%)** | 18.39±12.58 | 13.56±8.17 | 44.41±30.83 | 4.55 | 0.10 |
| **Health indicators** | **Co-exposure rate**^a^ | 55.33±9.83 | 30.53±16.89 | 19.18±12.30 | 9.64 | 0.008 |
| **Surface ENAD** | **Pathogen-related marker diversity (H’)** | 0.77±0.47 | 1.13±0.31 | 1.26±0.23 | 4.51 | 0.11 |
|  | **Pathogen-related marker richness** | 2.57±1.27 | 3.50±1.05 | 4.20±1.30 | 4.78 | 0.09 |

—“χ²”=chi-square value; “SD”=standard deviation—

^a^Co-exposure rate (described as *co-exposure degree index* in Perelló et al.-manuscript under review) [58]. This parameter uses information from serological analyses performed on wild boar serum samples collected from 18 study sites between 2022 and 2023. Antibodies against 12 pathogens were considered in the calculation of this parameter: *Brucella* spp., Aujeszky disease virus*, Erysipelothrix rhusiopathiae*, *Toxoplasma gondii*, *Mycoplasma hyopneumoniae*, porcine circovirus type 2, canine distemper virus, hepatitis E virus, *Coxiella burnetii*, Crimea-Congo hemorrhagic fever virus, epizootic hemorrhagic disease virus, *Mycobaterium tuberculosis* complex, and *Salmonella* spp. This parameter measures the weighted average intensity of pathogen co-exposure across study sites, where higher values indicate more wild boar exposed to multiple pathogens simultaneously.
