# Supplementary material for: Tracking pathogen-related markers with eDNA in natural areas: how environmental factors shape surveillance strategies
Source: Vet Res. 2026 Apr 28;57:90. doi: 10.1186/s13567-026-01746-6 (PMC13214320; doi:10.1186/s13567-026-01746-6)
Supplement: Supplementary file 5 — Additional file 5: Confusion matrix and statistics. This file contains the confusion matrix and statistics of predictive analyses. [file 13567_2026_1746_MOESM5_ESM.docx]

**Supplementary table 4**. Confusion matrix and statistics.

| **Confusion Matrix** | | | |
| --- | --- | --- | --- |
|  | **Reference** | | |
| **Prediction** | C1 | C2 | C3 |
| C1 | 6 | 1 | 0 |
| C2 | 1 | 5 | 0 |
| C3 | 0 | 0 | 5 |
| **Overall Statistics** | | | |
| **Accuracy** | | 0.89 | |
| **95%CI** | | (0.65, 0.99) | |
| **No Information Rate** | | 0.39 | |
| **P-Value [Acc>NIR]** | | 1.69e-05 | |
| **Kappa** | | 0.83 | |
| **Macro precision** | | 0.90 | |
| **Macro recall** | | 0.90 | |
| **Macro F1 score** | | 0.90 | |
| **Weighted precision** | | 0.89 | |
| **Weighted recall** | | 0.89 | |
| **Weighted F1 score** | | 0.89 | |
| **Statistics by Class** | | | |
| **Class** | **C1** | **C2** | **C3** |
| **Sensitivity** | 0.86 | 0.83 | 1 |
| **Specificity** | 0.91 | 0.92 | 1 |
| **PosPredValue** | 0.86 | 0.83 | 1 |
| **NegPredValue** | 0.91 | 0.92 | 1 |
| **Prevalence** | 0.39 | 0.33 | 0.28 |
| **DetectionRate** | 0.33 | 0.28 | 0.28 |
| **DetectionPrevalence** | 0.39 | 0.33 | 0.28 |
| **BalancedAccuracy** | 0.88 | 0.88 | 1 |

—C1=Cluster 1; C2=Cluster 2; C3= Cluster 3—
