# Supplementary material for: Tracking pathogen-related markers with eDNA in natural areas: how environmental factors shape surveillance strategies
Source: Vet Res. 2026 Apr 28;57:90. doi: 10.1186/s13567-026-01746-6 (PMC13214320; doi:10.1186/s13567-026-01746-6)
Supplement: Supplementary file 6 — Additional file 6: Prediction of the LOOCV using random forest method. This table show the accuracy of the predictive analyses across the different clusters. [file 13567_2026_1746_MOESM6_ESM.docx]

**Supplementary table 5**. Prediction of the LOOCV using random forest method.

| **Study area** | ***mtry*** | **Predicted** | **Observed** | **Cluster 1** | **Cluster 2** | **Cluster 3** | **Row index** |
| --- | --- | --- | --- | --- | --- | --- | --- |
| 1 | 10 | Cluster 1 | Cluster 1 | 0.85 | 0.14 | 0.01 | 1 |
| 2 | 10 | Cluster 1 | Cluster 1 | 0.93 | 0.07 | 0.00 | 4 |
| 3 | 10 | Cluster 1 | Cluster 1 | 0.90 | 0.09 | 0.004 | 3 |
| 4 | 10 | Cluster 1 | Cluster 1 | 0.95 | 0.04 | 0.01 | 2 |
| 5 | 10 | Cluster 1 | Cluster 1 | 0.89 | 0.11 | 0.01 | 5 |
| 6 | 10 | Cluster 3 | Cluster 3 | 0.38 | 0.11 | 0.50 | 14 |
| 7 | 10 | Cluster 2 | Cluster 2 | 0.17 | 0.80 | 0.03 | 10 |
| 8 | 10 | Cluster 1 | Cluster 1 | 0.76 | 0.23 | 0.01 | 6 |
| 9 | 10 | Cluster 3 | Cluster 3 | 0.03 | 0.48 | 0.49 | 15 |
| 10 | 10 | Cluster 2 | Cluster 2 | 0.01 | 0.88 | 0.12 | 11 |
| 11 | 10 | Cluster 2 | Cluster 1 | 0.36 | 0.59 | 0.05 | 7 |
| 12 | 10 | Cluster 3 | Cluster 3 | 0.07 | 0.26 | 0.67 | 16 |
| 13 | 10 | Cluster 2 | Cluster 2 | 0.10 | 0.74 | 0.16 | 12 |
| 14 | 10 | Cluster 1 | Cluster 2 | 0.98 | 0.02 | 0.00 | 8 |
| 15 | 10 | Cluster 3 | Cluster 3 | 0.02 | 0.22 | 0.76 | 17 |
| 16 | 10 | Cluster 2 | Cluster 2 | 0.01 | 0.76 | 0.23 | 13 |
| 17 | 10 | Cluster 2 | Cluster 2 | 0.34 | 0.46 | 0.19 | 9 |
| 18 | 10 | Cluster 3 | Cluster 3 | 0.01 | 0.26 | 0.74 | 18 |

—*mtry* parameter=number of variables randomly sampled as candidates at each split.—
