# Supplementary material for: Tracking pathogen-related markers with eDNA in natural areas: how environmental factors shape surveillance strategies
Source: Vet Res. 2026 Apr 28;57:90. doi: 10.1186/s13567-026-01746-6 (PMC13214320; doi:10.1186/s13567-026-01746-6)
Supplement: Supplementary file 7 — Additional file 7: Probability of each map pixel belonging to a cluster. This figure represents the probabilities of each map pixel belonging to a cluster. [file 13567_2026_1746_MOESM7_ESM.docx]

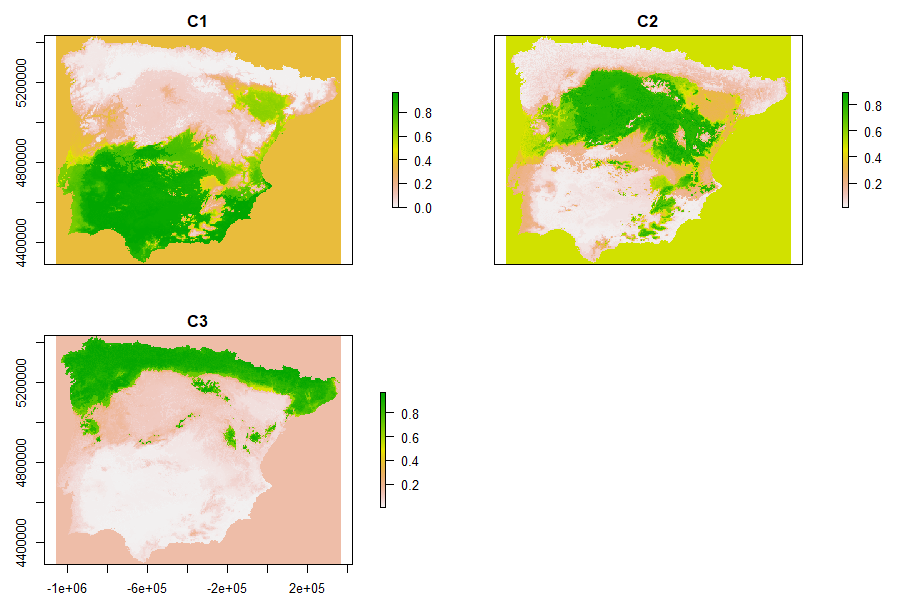


**Supplementary figure 2**. Probability of each map pixel belonging to a cluster. C1=Cluster 1; C2=Cluster 2; C3=Cluster 3.
