# Supplementary material for: Tracking pathogen-related markers with eDNA in natural areas: how environmental factors shape surveillance strategies
Source: Vet Res. 2026 Apr 28;57:90. doi: 10.1186/s13567-026-01746-6 (PMC13214320; doi:10.1186/s13567-026-01746-6)
Supplement: Supplementary file 8 — Additional file 8: Methods. This file describes in detail the PCR protocols and mammal community parameters. [file 13567_2026_1746_MOESM8_ESM.docx]

**SUPPLEMENTARY METHODS.**

**Detailed PCR protocols:**

All the direct, semi-nested and nested PCR protocols described for parasites were conducted on a 2720 Thermal Cycler (Applied Biosystems). Reaction mixes always included 2.5 U of MyTAQ™ DNA polymerase (Bioline GmbH, Luckenwalde, Germany) and 5–10 μl 5× MyTAQ™ Reaction Buffer containing 5 mM deoxynucleotide triphosphates and 15 mM MgCl2. Negative and positive controls were included in every PCR run. PCR amplicons were visualized on 2% D5 agarose gels (Conda, Madrid, Spain) stained with Pronasafe (Conda nucleic acid staining solutions. A 100 bp DNA ladder (Boehringer Mannheim GmbH, Mannheim, Germany) was used for the sizing of the obtained amplicons.

Real-time PCR of bacteria molecular markers were conducted in a final volume of 25 µL, containing QuantiFast Pathogen + IC (Qiagen, Hilden, Germany) 400 mM primers, and 200 nM probes. PCR amplification conditions were the following: Tag DNA polymerase activation at 95ºC for 5 minutes followed by 45 cycles of denaturation at 95ºC for 15 seconds and annealing at 60 to 61ºC for 30 to 60 seconds. The analysis using the Foodproof STEC Screening Lyokit was performed according to the manufacturer’s instructions.

Each reaction included an internal amplification control (IAC) to detect PCR inhibition. Samples were considered inhibited due to no amplification of the IAC in PCR reaction (the internal reaction control amplifies at 31 PCR cycles). All PCR procedures were performed under controlled laboratory conditions to minimize the risk of contamination. Preparation of the PCR reagent mix was carried out in a clean area, while the addition of sample DNA was performed in an intermediate area. Amplification in the thermocycler was conducted in a separate dirty area. This spatial separation of pre- and post-amplification steps ensured the reliability of the results throughout the process.

**Mammal community parameters:**

The parameters used to characterize the characteristics of the mammal community refer to the relative weight of the different species or groups of species within the community. This relative weight, as a proxy of a frequency-corrected relative abundance, has been obtained by multiplying the trapping rates (number of encounters per camera trap and day) by the detection frequency (% of cameras that detect that species), divided by the sum of these values ​​for all species detected at the study site (in %). From camera-trapping data, we defined “encounter” of a species as each independent photographic sequence within a time frame of 120 seconds. The formula for “relative weight” calculation is the following:

$${Relative weight (\%)}_{n}= \frac{{(trapping rate \times detection frequency)}_{n}}{\sum{(trapping rate \times detection frequency)}_{m}} \times100$$

where “n” is a species and “m” refers to all the species in each study site.

The photo-trapping data of the mammal community are taken from another related work by Perelló et al. at the same study sites.
